# Comparison of errors in ablation depth calculation after myopic femtosecond laser in situ keratomileusis in patients with different degrees of myopia: a prospective study

**DOI:** 10.1186/s12886-023-03200-z

**Published:** 2023-11-13

**Authors:** Yi-Dian Jiao, Zhi Yan, Tian-Qi Zhao, Hai-Xia Zhao

**Affiliations:** 1 Department of PRK Center, the Affiliated Hospital of Inner Mongolia Medical University, Tongdao North Street 1, Hohhot, 010050 China; 2 School of Medical Laboratory, Tianjin Medical University, Guangdong Road, Tianjin, 300203 China

**Keywords:** FS-LASIK, Pentacam, Errors in ablation depth calculation, Myopia

## Abstract

**Background:**

To investigate the difference between the predicted preoperative corneal ablation depth and the measured ablation depth for femtosecond laser in situ keratomileusis (FS-LASIK) in patients with different degrees of myopia, and to analyze the source of the difference.

**Methods:**

A total of 55 patients (109 eyes) were included in this study. Multiple logistics regression was applied to analyze the sources affecting postoperative refractive outcomes. The difference between the preoperative predicted corneal ablation depth and the 1-day postoperative ablation depth in patients with different degrees of myopia was explored using linear regression. Corneal biomechanical parameters influencing error in ablation depth calculation were examined using multiple linear regression.

**Results:**

One hundred and nine eyes were divided into low to moderate myopia (55 eyes, myopia of 6 D or less), high myopia (45 eyes, myopia ranging from 6 D to a maximum of 9 D), and very high myopia group (9 eyes, myopia greater than 9 D) based on preoperative refractive error (spherical equivalent). Postoperative visual outcomes were comparable among the three groups of patients, with no significant difference in uncorrected visual acuity (UCVA). We did find notable disparities in spherical equivalent (SE) and central corneal thickness (CCT) in patients with different degrees of myopia at 1 day postoperatively (all *p* < 0.001). Logistic regression analysis showed that error in ablation depth calculation was an independent risk factor for refractive outcomes one day after surgery (OR = 1.689, 95% CI: 1.366 – 2.089). There was a substantial discrepancy in error in ablation depth calculation at 1 day postoperatively between the three groups. The measured ablation depth of the laser platform was lower than the predicted ablation depth in the low to moderate myopia and very high myopia groups, but the opposite was true in the high myopia group. Pre-operative SE (*p* < 0.001) and corneal front minimum radius of curvature (Front Rmin) (*p* = 0.007) obviously influenced the error in ablation depth calculation.

**Conclusions:**

Error in ablation depth calculation values vary significantly between patients with different degrees of myopia and correlate highly with preoperative SE and Front Rmin. At the same time, the available evidence suggests that error in ablation depth calculation is an influential factor in postoperative refractive status, so it is imperative to control error in ablation depth calculation.

## Introduction

The global prevalence of myopia is almost 2 billion individuals (28.3% of the global population), which includes 277 million individuals (4.0%) with high myopia [1]. The prevalence is estimated to increase to 4.76 billion individuals (49.8%) for myopia and almost 1 billion individuals (9.8%) with high myopia by 2050 [1]. Increasingly, people are opting for refractive surgery to correct their vision, but the precision of refractive surgery needs to be further investigated.

Femtosecond laser flap-making excimer laser in situ keratomileusis (FS-LASIK) stands as the prevailing surgical method for addressing refractive errors, valued for its swift visual recovery, postoperative refractive stability, and strong track record of safety, efficacy, and predictability [2, 3]. FS-LASIK surgery operates on the fundamental principle of rectifying myopia by reshaping the anterior corneal surface via excimer laser ablation, with the reduction in central corneal thickness (CCT) serving as a measure of the depth of ablation [4]. In contemporary clinical practice, surgeons gauge the intraoperative ablation depth based on preoperative assessments and programming of the laser platform. This predicted value informs the surgeon’s actions during the procedure. It’s worth noting that over-thinning the residual stromal bed or excessive postoperative ablation may lead to corneal ectasia and failure to achieve optimal post-operative refractive outcomes [5, 6]. Previous studies to date have investigated the difference between the predicted and measured ablation depth of the laser platform at 1-month and 3-month after FS-LASIK [7–9], however, these investigations have often overlooked the potential influence of the postoperative recovery period on measurement results and have not probed deeper into the factors contributing to this divergence.

In this study, we utilized a rotating Scheimpflug camera (Pentacam, Oculus Optikgeräte, Wetzlar, Germany) 3D anterior segment analyzer to measure the CCT in FS-LASIK patients both before and 1 day after the surgery. Our primary aim was to explore the disparities between the predicted ablation depth and the measured ablation depth of the laser platform, shedding light on the factors that contribute to errors in ablation depth calculation.

## Materials and methods

### Study design and patients

In this prospective study, we collected data from 55 patients (109 eyes) who voluntarily underwent FS-LASIK treatment at the Affiliated Hospital of Inner Mongolia Medical University between October 2021 and May 2022. The patients were categorized into three groups based on their preoperative refractive error (spherical equivalent), specifically: low to moderate myopia (55 eyes, myopia of 6 D or less), high myopia (45 eyes, myopia ranging from 6 D to a maximum of 9 D), and a very high myopia group (9 eyes, myopia greater than 9 D). Exclusion criteria included (1) age < 18 years; (2) refractive error (spherical equivalent) increase greater than 0.50 D within the last year; (3) wore contact lenses within two weeks; and (4) history of other eye diseases or eye surgery. This study was approved by the Ethics Committee of the Affiliated Hospital of Inner Mongolia Medical University (No. WZ2022028).

### Data collection

All data were sourced from the Affiliated Hospital of Inner Mongolia Medical University. All patients underwent routine systematic ocular examinations before surgery, including slit lamp microscopy, intraocular pressure, uncorrected visual acuity (UCVA), best corrected visual acuity (BCVA), autorefractometry (Tomey RT-7000; Tomey Corp, Tokyo, Japan), corneal topography, visual corneal biomechanics analyzer (Corvis ST; Oculus Optikgeräte, Wetzlar, Germany), and fundus examination to exclude contraindications for FS-LASIK. Axial length (AL) was measured using high-frequency A-scan ultrasonography (PAC SCAN 300AP; Sonomed Escalon, USA). The patient’s corneal thickness was measured using Scheimpflug corneal thickness measurement (Pentacam HR; Oculus Optikgeräte, Wetzlar, Germany). All patients were reviewed for postoperative examinations at 1 day and 1 month after surgery, but this was not mandatory. UCVA measurement, corneal topography, autorefractometry, and slit lamp examination at each postoperative follow-up visit. The measured ablation depth is the difference between the preoperative and 1-day postoperative Pentacam measurements, and the predicted ablation depth is the theoretical ablation depth calculated by the laser platform system software. The error in ablation depth calculation is defined as the difference between the preoperative predicted ablation depth and the measured ablation depth (Errors in ablation depth calculation = Measured ablation depth – Predicted ablation depth). In this study, all preoperative and postoperative examinations were performed by the same skilled operator, so that the influence of human factors on the accuracy of the examination results could be excluded.

FS-LASIK was performed using nanofocus femtosecond (ziemerFemto LDV Z4, Ziemer, Swit) flap preparation and ablation with an excimer WaveLight laser (EX500, Allegretto, Germany). The ablation zone was 6.50 mm in diameter for all participants, the flap diameter and depth were set at 9 mm and 110 μm respectively, and the ablation zone was programmed to have a residual corneal stromal bed depth of ≥ 300 μm for all patients. All patients underwent surgery performed by the same skilled surgeon.

### Statistical analysis

The Kolmogorov-Smirnov test was used to determine the normal distribution of continuous data. For normally distributed data, we expressed results as the mean ± SD, while for non-normally distributed data, we presented results as the median (interquartile range). To compare continuous variables, we employed either a one-way analysis of variance (ANOVA) or Kruskal-Wallis H tests, depending on the data’s distribution. Categorical variables were reported as n (%) and were subjected to analysis using the chi-square test. To develop a risk model for errors in ablation depth calculation, we utilized multivariate logistic regression. Additionally, we conducted multiple linear regression to explore the relationship between the error and each parameter. All analyses and graphs were performed with SPSS 23.0 (IBM, Armonk, NY, USA) and GraphPad Prism 6.0 (GraphPad Software), and *p* < 0.05 was regarded as statistically significant.

## Results

### Characteristics of the participants

**Fig. 1:**
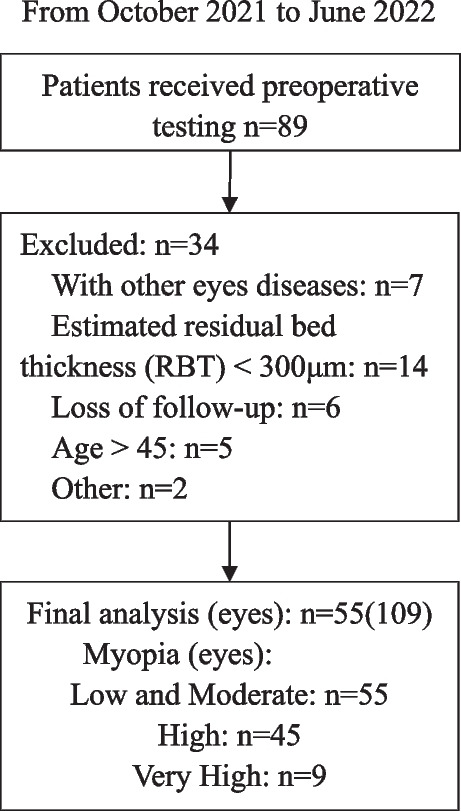
A flowchart of the patient selection process

Figure 1 is a flow chart of the participant selection process. We included 55 patients (109 eyes) in this study. The characteristics of the participants are listed in Table 1. Due to the low number of patients with low myopia undergoing FS-LASIK surgery in the region, we artificially divided all participants into three groups, including Low and Moderate Myopia, High Myopia, and Very High Myopia. The AL was significantly longer in the Very High Myopia patients than in the other two groups. This result was also visible in the preoperative spherical equivalent (SE).

**Table 1:** Demographic characteristics and ocular parameters of the participants

Characteristics	Total (*n* = 55/109)	Myopia			F/H/χ^2^	*p*
		Low and Moderate (*n* = 55)	High (*n* = 45)	Very High (*n* = 9)		
Age (years)	25 (22, 32)					
IOP (mmHg)	15.64 ± 2.57	16 (14, 18)	15 (14, 17)	18 (14, 18)	1.248	0.536
AL (mm)	24.86 (24.45, 25.63)	24.58 ± 0.71	25.28 ± 0.87	26.29 ± 0.64	23.50	< 0.0001
Pre-BCVA = 20/20 (%)	106 (97.2)	55 (100)	43 (95.6)	8 (88.9)	4.387	0.112
Pre-SE (D)	–6.25 (–5.00, −7.50)	–5.00 (–4.00, −5.50)	–7.00 (–6.50, −7.88)	–9.75 (–9.38, −10.13)	86.73	< 0.0001

Data are presented as mean ± SD or median (interquartile range), compared with chi-square test, one-way analysis of various (ANOVA) or Kruskal–Wallis H tests. *IOP *Intraocular pressure, *AL *Axial length, *Pre-BCVA *Pre-operative best corrected visual acuity, *Pre-SE *Pre-operative spherical equivalent

### Postoperative refractive status and effective factors

**Fig. 2:**
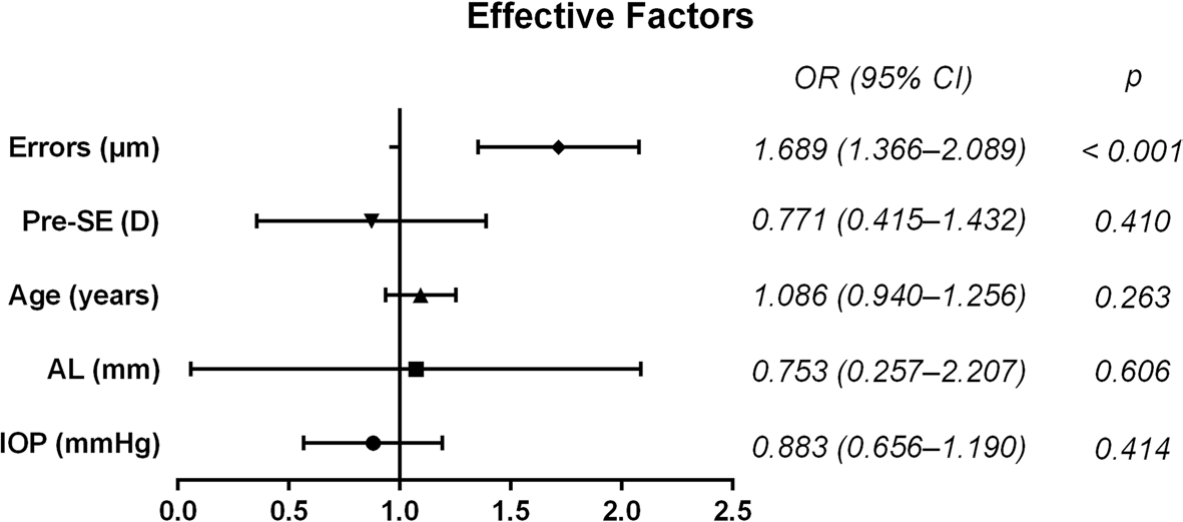
Logistic regression analysis of 1-day post operation SE. Errors, error in ablation depth calculation; Pre-SE, pre-operative spherical equivalent; AL, axial length; IOP, intraocular pressure

Table 2 lists the UCVA and SE for the three groups of patients at 1 day and 1 month postoperatively, and there was no significant difference in postoperative UCVA between the three groups of patients. The SE of patients with different degrees of myopia was significantly different at both postoperative times. Figure 2 shows a logistic regression analysis of the risk factors of postoperative refractive status in FS-LASIK patients. Error in ablation depth calculation (OR = 1.689, 95% CI: 1.366 – 2.089, *p* < 0.001) was an independent risk factor for postoperative refractive status.

**Table 2:** Postoperative refractive status

Groups	n	UCVA = 20/20		SE (D)	
		1-day post operation (%)	1-month post operation (%)	1-day post operation	1-month post operation
Low and Moderate Myopia	55	55 (100)	54 (98.2)	0.41 ± 0.42	0.20 ± 0.03
High Myopia	45	41 (88.9)	43 (95.6)	0.29 ± 0.61	0.06 ± 0.21
Very High Myopia	9	8 (88.9)	8 (88.9)	–0.53 ± 0.38	–0.22 ± 0.32
F/χ^2^		5.422	2.020	13.225	9.840
*p*		0.066	0.364	< 0.001	< 0.001

Data are presented as mean ± SD, compared with chi-square test or one-way analysis of various (ANOVA). *UCVA *Uncorrected visual acuity, *SE *Spherical equivalent

### Differences in errors in ablation depth calculation between patients with different degrees of myopia

**Fig. 3:**
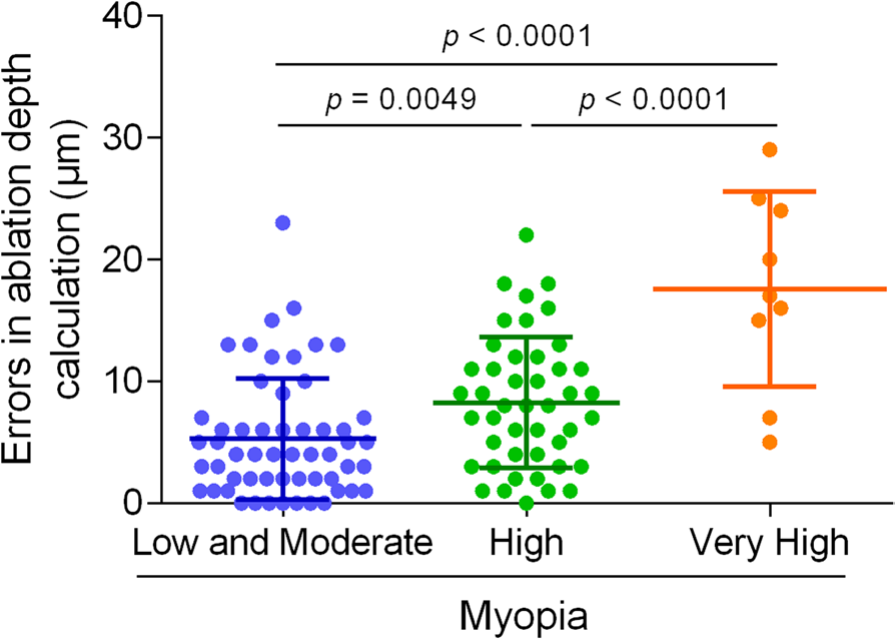
Scatter plots of errors in ablation depth calculation for patients with different degrees of myopia

**Fig. 4:**
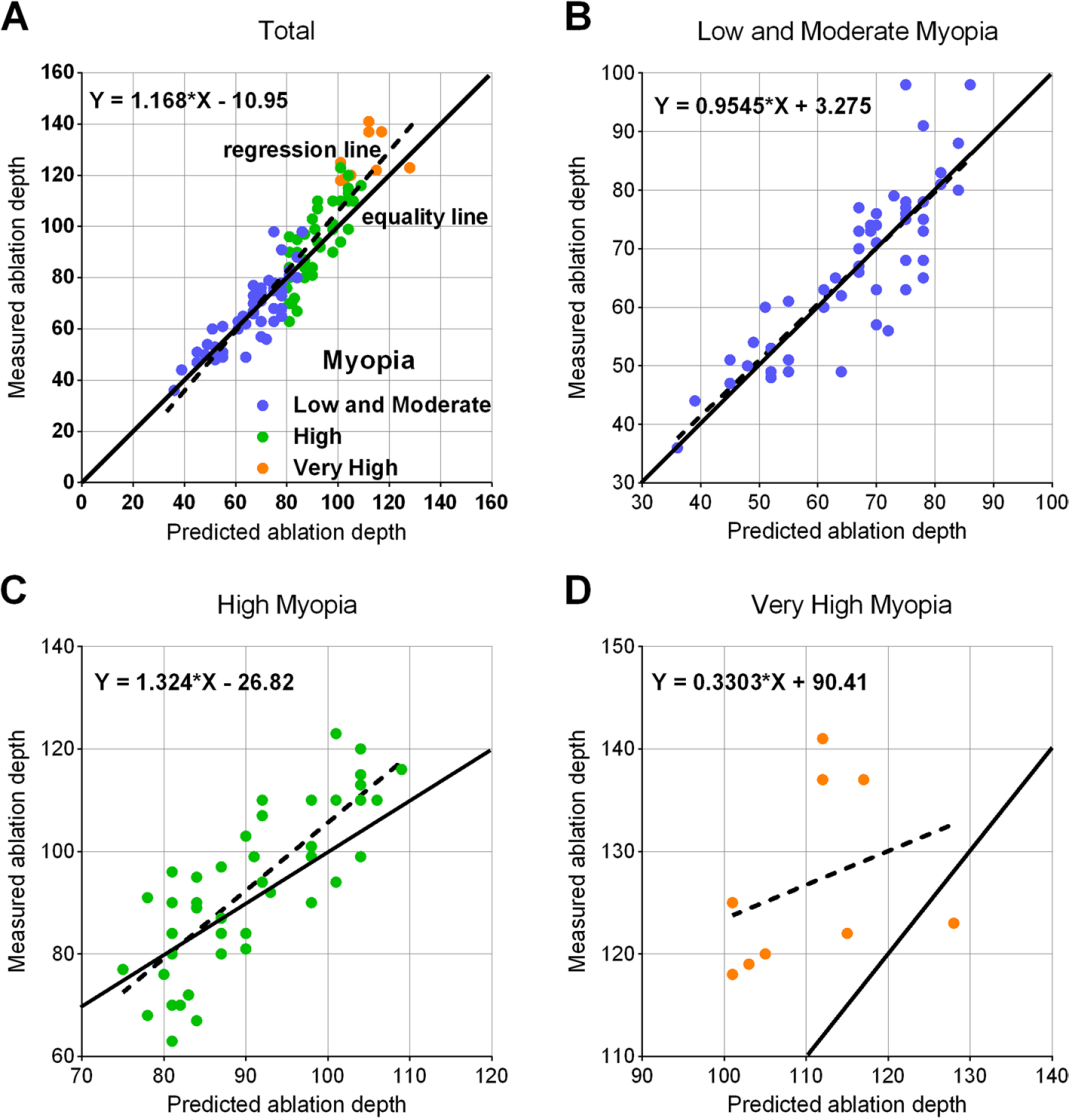
Comparison of predicted ablation depth and measured ablation depth in different refractive groups

Figure 3 shows scatter plots of errors in ablation depth calculation for the three groups of patients. There were significant differences in errors in ablation depth calculation between the three groups of patients. Specifically, the patients with very high myopia demonstrated significantly higher errors in ablation depth calculation compared to the other two groups. This suggests that the risk of error in ablation depth calculation is positively associated with the degree of myopia. Table 3 lists the differences between Measured ablation depth and Predicted ablation depth. It is noteworthy that while the mean preoperative central corneal thickness (CCT) did not significantly differ among the three groups (536.55 ± 31.20 μm, 539.18 ± 25.37 μm, and 551.11 ± 21.16 μm, respectively; *p* = 0.359), significant differences were observed between the measured and predicted ablation depths across all three groups (*p* < 0.001). Figure 4 A presents a scatterplot illustrating the relationship between predicted ablation depth (x-axis) and measured ablation depth (y-axis) for various myopia subgroups. Notably, a correlation between predicted and measured ablation depths is observed in the entire participant population (b = 1.168, *p* < 0.0001). Further examination reveals that in the low and moderate myopia group (Fig. 4B) and the very high myopia group (Fig. 4C), the measured ablation depth by the laser platform was lower than the predicted ablation depth. Conversely, in the high myopia group (Fig. 4D), the measured ablation depth exceeded the predicted ablation depth.

**Table 3:** Differences between measured ablation depth and predicted ablation depth

Groups	CCT (µm)				Errors in ablation depth calculation (µm)
	Preoperative	1-day post operation	Measured ablation depth	Predicted ablation depth	
Low and Moderate Myopia	536.55 ± 31.20	469.60 ± 34.62	66.95 ± 13.65	66.84 ± 12.21	5.29 ± 4.97
High Myopia	539.18 ± 25.37	446.53 ± 26.43	92.64 ± 15.32	90.2 ± 9.28	8.27 ± 5.36
Very High Myopia	551.11 ± 21.16	424.22 ± 15.32	126.89 ± 8.91	110.44 ± 8.95	17.56 ± 8.00
F	1.035	12.679	89.976	97.658	20.555
*p*	0.359	< 0.001	< 0.001	< 0.001	< 0.001

Data are presented as mean ± SD, compared with one-way analysis of various (ANOVA). Errors in ablation depth calculation = (Measured ablation depth – Predicted ablation depth). *CCT *Central corneal thickness

### Analysis of sources of error in ablation depth calculation

As our results show that there are errors in ablation depth calculation in the FS-LASIK, we felt it necessary to analyze their origin. Multiple linear regression models of the influence of patients’ ocular parameters on errors in ablation depth calculation are shown in Table 4. It can be observed that errors in ablation depth calculation were significantly higher in those with higher pre-SE (*p* < 0.001) and larger corneal front minimum radius of curvature (Front Rmin) (*p* = 0.007).

**Table 4:** Multiple linear regression of potential factors related to errors in ablation depth calculation

	B	SE	*p*
Pre-SE (D)	1.850	0.291	< 0.001
Pre-CCT (mm)	0.10	0.019	0.584
Front Rper (mm)	0.002	0.007	0.823
Front Rmin (mm)	11.242	4.070	0.007
Back Rper (mm)	–8.212	4.583	0.076
Back Rmin (mm)	1.149	4.477	0.798

*B *Unstandardised coefficient, *SE *Standard error, *Pre-SE *Preoperative spherical equivalent, *Pre-CCT *Preoperative central corneal thickness. Rper, center of zone radius between the 7 and 9 mm ring; Rmin, minimum radius of curvature

## Discussion

In this study, to verify the accuracy of laser estimates of post-operative corneal ablation depth, the predicted ablation depths were compared with the actual measured ablation depths from the laser platform. Notably, while previous studies have examined the disparity between predicted and measured ablation depths in the context of FS-LASIK refractive surgery, these assessments were typically conducted at the 1-month and 3-month postoperative marks. Regrettably, this approach overlooks the potential impact of the postoperative recovery period on the measurement results [7–9]. To mitigate the influence of time-related factors on our measurements, we opted to evaluate errors in ablation depth calculation just 1 day after surgery which allowed us to provide a more immediate and potentially less variable assessment of the accuracy of the laser’s ablation depth predictions [10, 11]. Our evidence shows that errors in ablation depth calculation values differ significantly between patients with different degrees of myopia and are highly correlated with Preoperative SE and Front Rmin.

We observed significant differences in postoperative refractive errors among the low to moderate myopia, high myopia, and very high myopia groups at both 1 day and 1 month postoperatively (all *p* < 0.001). In cases of very high myopia and high myopia, it is often advantageous to retain a certain degree of myopia postoperatively to avoid overcorrection, as removing excessive corneal tissue can lead to potential complications such as corneal ectasia [12, 13]. On the contrary, patients with moderate to low myopia often experience some degree of postoperative hyperopia, which contributes to better long-term visual outcomes without compromising the cornea’s biomechanical stability [14]. Previous studies have indicated a slight decrease in intraocular pressure (IOP) following FS-LASIK, primarily due to changes in corneal thickness resulting from flap creation and other biomechanical factors [15, 16]. Although these changes in corneal biomechanics can affect IOP measurements, they are usually temporary [17]. However, it’s important to note that our study did not monitor postoperative IOP changes, which is a limitation. Immediately afterward, we conducted multiple logistic regression to analyze the factors that could impact the difference in postoperative spherical equivalent. Our findings revealed a significant influence of errors in ablation depth calculation on postoperative refractive outcomes (OR = 1.689; 95% CI: 1.366 – 2.089; *p* < 0.001). This is consistent with the study by Kanellopoulos et al. [18] that actual objective stromal depth reduction following myopic LASIK correlates well with the attempted versus achieved refractive change.

Many studies have found differences between the measured ablation depth 1 month post FS-LASIK and the preoperative predicted corneal ablation depth [8–19], and our study confirmed this 1 day postoperatively. The measured ablation depth of the laser platform ablation 1 day after FS-LASIK in the 109 eyes included in this study was 2.49 ± 9.54 μm higher than the predicted value (*p* = 0.008). Although there was an error, we consider this to be clinically negligible [8]. We also assessed the difference between the measured corneal ablation depth at 1 day postoperatively and the preoperative predicted corneal ablation depth in patients with different degrees of myopia. Notably, the error in ablation depth calculation at 1 day postoperatively exhibited a sequential increase in the three groups: low to moderate myopia (5.29 ± 4.97 D), high myopia (8.27 ± 5.36 D), and very high myopia (17.56 ± 8.00 D) (*p* < 0.001). Linear regression analysis demonstrated distinct patterns: (Y = 0.3303X + 90.41, *p* = 0.3833), the measured ablation depth on the laser platform was lower than the predicted ablation depth, while for high myopia (Y = 1.324*X – 26.82, *p* < 0.0001), it was the opposite. Interestingly, our findings regarding over-ablation in eyes with higher degrees of myopia correction differed from the study by Savini et al. [20], this discrepancy may be attributed to the relatively small sample size in our very high myopia group.

To further explore the sources of errors in ablation depth calculation, we employed a multiple linear regression model. Our analysis revealed that Preoperative SE (B = 1.850, *p* < 0.001) and Front Rmin (B = 11.242, *p* = 0.007) exerted significant and positive influence on the error in ablation depth calculation. However, it’s important to note that our study had certain technical limitations, including the omission of several corneal biomechanical parameters. We encourage future research endeavors to expand upon our findings and delve deeper into the underlying sources of errors in ablation depth calculation.

We acknowledge several limitations of this study: firstly, we included measurements from the contralateral eye of the same patient, which may potentially affect the results due to the association of corneal values between the eyes (intraclass correlation coefficients > 0.90). This is even though when only one eye was used per patient, it was consistent with the results of the present study. Secondly, the sample size in our study is relatively small and meaningful comparison may be difficult using this study’s data. Necessitating further observations with larger sample sizes across all subgroups to enhance the robustness of our findings. Thirdly, this was a single-center study conducted in northwest China, which may have biased the results. Fourthly, some risk factors, such as keratometry and postoperative IOP were not investigated.

## Conclusions

In our study, the Pentacam measurements and the predicted results of the laser platform for ablation depth correlated well, but discrepancies still existed. Therefore, it is important to consider preoperative refraction when entering patient data in the laser software preoperatively to improve the accuracy of true estimates of ablation depth and remaining stromal layer depth. In addition, multiple linear regression revealed Preoperative SE and Front Rmin as the main sources of error in ablation depth calculation. Therefore, we believe that Preoperative SE and Front Rmin should be used as parameters for the surgeon to consider the patient’s postoperative refractive outcome. In the clinical application of FS-LASIK, whether the refractive surgeon needs to set a greater predicted ablation depth to correct the target refraction for patients with high myopia is subject to further clinical study.

## Data Availability

The datasets used and/or analyzed during the current study are available from the corresponding author on reasonable request.

## Abbreviations

FS-LASIK: Femtosecond laser in situ keratomileusis
SE: Spherical equivalent
CCT: Central corneal thickness
UCVA: Uncorrected visual acuity
BCVA: Best corrected visual acuity
AL: Axial length
ANOVA: Analysis of variance
Rper: The center of zone radius between the 7 and 9 mm ring
Rmin: The minimum radius of curvature
